# A Breastfeeding Relaxation Intervention Promotes Growth in Late Preterm and Early Term Infants: Results from a Randomized Controlled Trial

**DOI:** 10.3390/nu14235041

**Published:** 2022-11-27

**Authors:** Sarah Dib, Jonathan C. K. Wells, Simon Eaton, Mary Fewtrell

**Affiliations:** Great Ormond Street Institute of Child Health, University College London, London WC1N 1EH, UK

**Keywords:** preterm, breastfeeding, maternal stress, growth, parent-offspring signaling, late preterm, early term, breast milk composition, relaxation

## Abstract

Breastfeeding involves signaling between mother and offspring through biological (breast milk) and behavioral pathways. This study tested this by examining the effects of a relaxation intervention in an understudied infant population. Breastfeeding mothers of late preterm (34^0/7^–36^6/7^ weeks) and early term (37^0/7^–38^6/7^ weeks) infants were randomized to the relaxation group (RG, n = 35), where they were asked to listen to a meditation recording while breastfeeding from 3 weeks post-delivery, or the control group (CG, n = 37) where no intervention was given. Primary outcomes-maternal stress and infant weight-were assessed at 2–3 (baseline) and 6–8 weeks post-delivery. Secondary outcomes included infant length, infant behavior, maternal verbal memory, salivary cortisol, and breast milk composition. Infants in the RG had significantly higher change in weight-for-age Z-score compared to those in CG (effect size: 0.4; 95% CI: 0.09, 0.71; *p* = 0.01), and shorter crying duration [RG: 5.0 min, 0.0–120.0 vs. CG: 30.0 min, 0.0–142.0; *p* = 0.03]. RG mothers had greater reduction in cortisol (effect size: −0.08 ug/dL, 95% CI −0.15, −0.01; *p* = 0.03) and better maternal verbal learning score (effect size: 1.1 words, 95% CI 0.04, 2.1; *p* = 0.04) than CG mothers, but did not differ in stress scores. A simple relaxation intervention during breastfeeding could be beneficial in promoting growth of late preterm and early term infants. Further investigation of other potential biological and behavioral mediators is warranted.

## 1. Introduction

Of the 11% of infants born prematurely each year, around 74% are born late preterm (34^0/7^–36^6/7^ weeks) [1]. Compared to their term counterparts, late preterm infants (LPI) have a significantly higher risk of mortality and short- and long-term morbidity [2]. Their mothers also experience higher levels of anxiety, depression and stress [3]. Early term infants (ETI; 37^0/7^–38^6/7^ weeks) are another group who have higher risk of morbidity and mortality compared to those born later [4,5]. The exact nutrition requirements of LPI and ETI to support optimal growth are unknown; they are likely to be higher than those of full-term infants but not so high that they cannot be met by unsupplemented breast milk [6]. Therefore, current recommendations strongly support breastfeeding for these infants given that breast milk contains a myriad of components that are particularly beneficial to infants born earlier. It is also well recognized that breastfeeding in general is an optimum source of nutrition that confers short-term and long-term benefits for both mothers and infants [7]. Despite this, multiple studies have shown that LPI and ETI are less likely to be breastfed [8,9,10].

Results from multiple systematic reviews which include LPI and ETI demonstrate that there are very few breastfeeding interventions targeting this population and they have mainly focused on providing additional support or education before discharge [11,12,13]. Several studies have investigated relaxation interventions during breastfeeding, although not specifically in LPI or ETI [14,15,16]. These studies reported a reduction in maternal stress, an increase in breast milk intake/yield and an improvement in infant weight gain. It is scientifically plausible that a relaxation intervention could result in an improvement in breastfeeding outcomes and infant growth and development. From a physiological perspective, mothers who are less stressed may produce more milk and/or milk of higher macronutrient concentration, due to the reduction of stress-related hormones (cortisol) and thus reduced inhibition of breastfeeding-related hormones (oxytocin and prolactin). Less stressed mothers may also transfer different bioactive components through breast milk which may in turn influence infant behavior and growth. Behaviorally, stress could interfere with the mother’s engagement with the infant, where maternal disengagement or anxious over-involvement could negatively affect the mother-infant relationship and maternal sensitivity to infant cues.

From an evolutionary perspective, reducing maternal stress could ease mother-infant tension over maternal metabolic resources [17], and thus increase maternal investment in the offspring. Life history theory [18], which explains variation in the timing of growth, developmental rate, reproduction and death of living organisms, and parent-offspring conflict theory [17] are two theories that could help design maternal/child health interventions. They both take into consideration that variation in parental resources and experiences can differentially influence infant feeding decisions and signals transmitted to the offspring that impact infant growth and development. For instance, life history theory can help explain why mothers of lower income, lower education, higher stress in early life, shorter height, and/or earlier maturation might be more likely to reproduce early and often, and might therefore have fewer resources to invest in each offspring [19,20]. Parent-offspring conflict theory can help explain the tension and the ongoing negotiation between parents and offspring over the extent to which maternal nutritional resources should be invested in each offspring. By taking into consideration these concepts, we planned to assess outcomes that broadly investigate maternal phenotype as a marker of trade-offs between investing in the infant versus maintaining maternal phenotype. This may shed new light on issues that are discussed in literature (such as the impact of maternal stress on infant growth, or maternal memory) but where there is a lack of a clear framework to understand them.

Overall, there is a need for interventions that address the complex biological, psychological and behavioral variables involved in breastfeeding, especially those specific to late preterm and early term infants. Thus, the aim of this study was to investigate maternal investment through breastfeeding from biological and evolutionary perspectives using an experimental study design in this vulnerable group of mothers and infants.

## 2. Materials and Methods

### 2.1. Study Participants and Design

**Figure 1:**
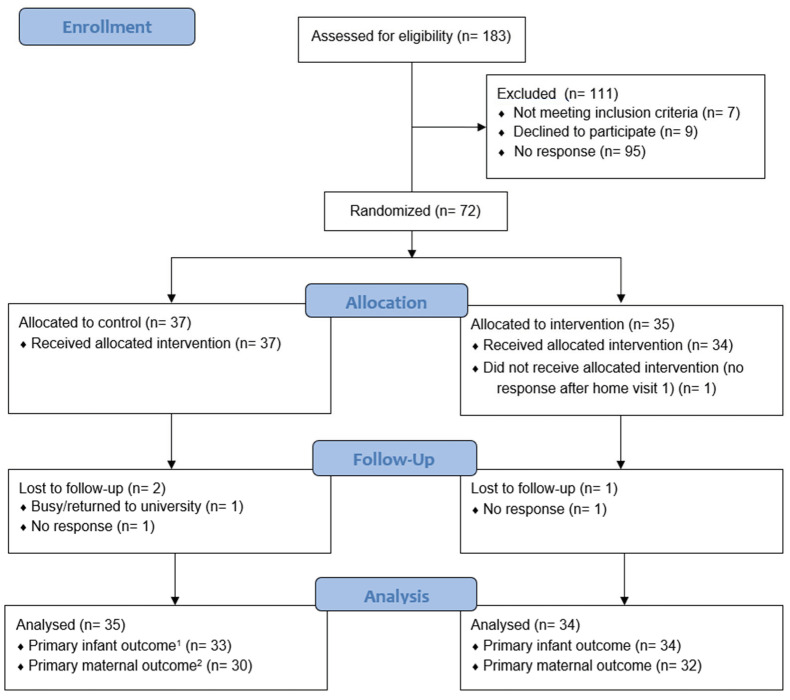
CONSORT flow diagram. ^1^ Primary infant outcome is change in weight-for-age z-score at 6–8 from baseline (2–3 weeks). ^2^ Primary maternal outcome is change in stress score at 6–8 weeks from baseline (2–3 weeks).

This was a randomized controlled trial using a breastfeeding meditation audio for mothers of late preterm and early term infants. Building upon the findings of a similar previous trial in full term infants [16], the hypothesis was that participants who listened to the intervention would become less stressed and their infants would gain more weight. The full study protocol was published elsewhere [21]. Briefly, mothers of healthy infants of 34^0/7^ to 38^6/7^ gestational weeks were identified and screened before discharge from three hospitals (Royal Free, Barnet and University College London Hospitals) in London. Mothers were eligible if they had a singleton pregnancy, intended to breastfeed for at least 6 weeks, spoke and understood English, did not smoke, were free of serious illness, and did not have a prior breast surgery that could interfere with breastfeeding. Overall, 183 participants were assessed for eligibility between January 2019 and January 2021. Of these, 72 provided informed consent and were randomized to the relaxation group (RG), where the audio was sent to participants on their mobile phones (or on an MP3 player, according to preference), or to the control group (CG) where no intervention was given (Figure 1). The study was ethically approved by the National Research Ethics Service and the Health Research Authority in the UK (18/LO/1835) and registered with ClinicalTrials.gov (NCT03791749). The CONSORT checklist is provided in Supplementary Table S1.

Based on the results of a previously published pilot study [22], a guided-imagery breastfeeding meditation was selected as the method of relaxation in this study. The 11-min audio was recorded by a mental health nurse with experience in hypnobirthing and meditation. Participants in the RG were encouraged to use the meditation, while breastfeeding, preferably using head/earphones, as many times as they wanted, but at least once a day for the first 2 weeks starting at baseline (2–3 weeks post-delivery). They were also asked to record the use of the meditation in a log. Participants in both groups received standard advice on infant feeding. They were also able to seek breastfeeding advice from the lead researcher and were referred to their midwife/health visitor or their local baby clinic if needed.

### 2.2. Randomization and Masking

The randomisation was stratified by gestational age (34 weeks, 35–36 weeks, 37–38 weeks) and parity (primiparous, multiparous). The randomisation schedule was produced by computer in blocks of permuted length (2–4–6). Assignment was prepared by M.F., who did not have any contact with the participants, and each assignment was placed in a sealed opaque envelope. The assignment was revealed after data collection was completed at the baseline home visit, to remain blinded to the assignment throughout the first home visit. After the assignment was revealed, if the participants were in the intervention group, the use of the meditation audio was explained and their preference for receiving the meditation was noted (by phone, YouTube link, MP3 player). Due to the nature of the intervention, it was not possible to blind the intervention to the participants and to the researcher involved in data collection. However, measures were taken to avoid issues introduced by non-blinding. First, the primary infant outcome was measured using an ‘objective’ method (weight) to reduce the chance of bias by the researcher and participant. To reduce the bias among participants and contamination between the control group and intervention group, which was likely due to the easily accessible intervention, participants were not informed about the randomisation process until the end of the study when the study results were communicated. Lastly, the lab analysis of saliva and breast milk samples was done masked to the intervention.

### 2.3. Data Collection

Home visits were conducted at 2–3 weeks (home visit 1; HV1) and 6–8 weeks (home visit 2; HV2) post-delivery by the lead researcher to collect data from both groups. At the baseline visit (HV1), demographic information such as income, education, ethnicity, and marital status was collected. At both visits, the primary maternal outcome, maternal stress, was assessed using the Perceived Stress Scale (PSS), where higher scores indicate higher stress [23]. The primary infant outcome, infant weight, was also measured in duplicate using a digital infant weighing scale (Seca 354, Japan) at both home visits, with the infant unclothed, without a diaper and before feeding whenever possible.

Secondary outcomes were also collected at home visits. Recumbent length was measured in duplicate at both visits using an infant length measuring mat (Rollameter, Raven). Infants were placed in supine position with their head against the headboard and the base of their feet against the vertical plate. In a few cases, it was not possible to take the infant’s weight or length; for example, the baby was distressed. In these instances, weight/length measured by the midwife, health visitor or GP was recorded from the baby’s personal child health record (red book). The average of the duplicate measurements was calculated and converted into a z-score using the Intergrowth 21st Postnatal Growth Standard calculator [24] which used weight, gestational age, age at home visit, and infant sex.

Participants completed the Edinburgh Postnatal Depression Scale (EPDS) [25], Maternal Responsiveness Questionnaire [26], Maternal Attachment Index [27], and Baby Eating Behavior Questionnaire [28] to assess maternal depression, responsiveness to infant cues, mother-infant attachment, and infant appetite, respectively. Infant crying, sleeping and fussiness durations were evaluated using a 3-day infant behavior diary [29], where the mother was asked to indicate whether the infant was crying, sleeping, feeding, being fussy, or awake and content during 15-min segments over 72 h. Maternal verbal memory was assessed using Rey’s Auditory Verbal Learning Test, which consists of 5-word recall trials and one delayed word recall trial. The outcomes of the test include the following based on previously described criteria [30]: 1. *Immediate recall*: sum of correct responses after first 5 trials, 2. *Verbal learning*: difference in number of correct responses after Trial 5 and Trial 1, and 3. *Verbal forgetting*: difference between correct responses after the delayed recall and Trial 5. Lastly, to estimate breast milk intake, participants were asked to complete 48-h test-weighing and a 24-h breastfeeding diary where they recorded the timings and durations of feeds, the feeding method (bottle vs. direct), and the type of feed (breast milk vs. formula milk). To estimate the volume of breast milk from the duration of breastfeeding per day, we calculated the average ml/min using data from mothers who completed at least 24 h of test-weighing in addition to 24 h of breastfeeding diary. Duration of breastfeeding for all participants who completed the 24-h breastfeeding diary was then converted to breast milk volume by multiplying it by 3.1 mL/min (95% CI 1.2, 4.9). A correction of 3% was made to account for insensible water loss [31].

Maternal saliva samples were collected at both visits using the passive drool method where the participant transfers saliva into a 1.5 mL collection tube with a Saliva Collection Aid (Salivette, Sarstedt, Germany). Participants were asked not to consume caffeine or brush their teeth for one hour before the saliva collection. They were also asked to rinse their mouth with water 10–15 min before saliva collection. Samples were collected before breastfeeding, whenever possible, and at a standardized time of day (10 am–12 pm). They were then stored in a travel ice bag and transferred to University College London, within ~1–2 h (depending on the transportation time) to be stored at −80 °C until analysis. A competitive immunoassay kit (salivary cortisol ELISA kit; Salimetrics) was used to analyze the samples in duplicate. The 1.5 mL tubes were thawed, vortexed, then centrifuged at 1500×*g* for 15 min to remove mucins and other materials that might interfere with the assay. Twenty-five μL of standards, controls, and saliva samples were pipetted into appropriate wells, then 200 μL of the diluted enzyme conjugate was added. After incubating at room temperature and washing the plate using the wash buffer and a plate washer, 200 μL of TMB Substrate was added. Lastly, the plate was incubated in a dark place, the stop solution was added, and the plate was read in a plate reader using a 450 nm wavelength filter. Duplicates with a variance greater than 15% were not included in the analysis due to the risk of lab measurement error.

Breast milk samples were also collected at both visits using a standardized procedure described elsewhere and designed to minimise the variability in breast milk composition as a result of the time of day, breast fullness and time since previous feed [21]. The collected tubes were placed in a travel ice bag and transferred within 1–2 h (depending on transportation time) to be stored in a −80 °C freezer at University College London until analysis. In some instances, it was not possible to deliver the milk immediately to the −80 °C freezer, in which case it was stored at around −20 °C, and transferred as soon as possible (within one week) to the −80 °C storage facility. One or two 5 mL tubes for each participant were used for duplicate analysis of macronutrients using the MIRIS Human Milk Analyser (Miris, Sweden).

### 2.4. Changes to Study Methods Due to COVID-19 Pandemic

The study was interrupted from March 2020 to September 2020 due to COVID-19 restrictions. However, after resuming the study in September, changes in the data collection procedures were implemented in line with available guidance at the time to ensure the safety of the hospital staff, participants and researcher. Follow-up visits (2–3 and 6–8 weeks post-delivery) were no longer conducted face-to-face and instead were conducted over the telephone. If the infants were not measured by the GP, midwife, or health visitor during these two time periods, weighing scales and infant length meters were delivered (in a socially distanced manner) to mothers’ homes with written and verbal instructions on how to conduct the measurements. Breast milk collection equipment such as tubes and breast pumps (if needed) were sent by post with instructions. Questionnaires were sent by post or electronically depending on preference. All materials were accompanied by a step-by-step study procedure guide. These changes impacted 22/72 (31%) participants who were recruited during the pandemic.

### 2.5. Data Analysis

A power calculation was used to estimate the sample size based on a previous study that was able to detect significant and clinically important differences in weight z-score (σ = 0.8, d = 0.63) and stress score (σ = 4.4, d = 3.51) in response to a similar breastfeeding meditation audio [16]. Therefore, a sample of 52 mothers would be required to detect a significant difference in weight z-score at 5% with 80% power (σ = 0.8, d = 0.63). Our sample of breastfeeding mothers of late preterm and early term infants were expected to be more stressed and thus might have lower reductions in stress in response to the relaxation intervention compared to the mothers of healthy full-term infants involved in the previous study. Consequently, a mean difference of 3 in PSS score was used instead of 3.51 and a sample of 68 mothers would be needed to detect a difference in stress score significant at 5% with 80% power (σ = 4.4, d = 3). Allowing for drop-outs, the target sample size was 80 mother-infant pairs.

Data were analyzed using SPSS v26.0. The main analysis of the randomized controlled trial results was on an intention-to-treat basis for all subjects with outcome data available. For the primary outcomes, the changes in stress score and weight-for-age z-score from baseline to 6–8 weeks were compared between groups using independent sample *t*-test. Similarly, for the secondary outcomes, differences at HV2 and changes between baseline and HV2 between CG and RG in milk composition, milk supply, maternal cognitive function, maternal salivary cortisol, EPDS score, and mother–infant attachment were analyzed using independent sample *t*-test. For infant behavior, especially infant crying, the data was skewed and thus Mann-Whitney U test was used. The effect of gestational age and infant sex was evaluated using linear regression for variables with significant differences between groups. Further exploratory analyses were performed to assess the interactions between intervention group assignment and recruitment during the COVID-19 pandemic, infant’s gestational age, and infant’s sex on the primary and secondary outcomes. Results were considered statistically significant at *p* < 0.05.

## 3. Results

A total of 72 breastfeeding mothers were randomized (n for RG = 35; CG = 37; Figure 1). Eighty-one percent versus 94% of randomized participants had complete data for PSS change and 89% versus 97% for infant weight change, but the difference was not significant (*p* = 0.2 for both). There were no significant differences in baseline characteristics between those who had missing versus complete primary outcome data. There were also no differences between RG and CG in baseline characteristics (Table 1) or hospital practices experienced (Table 2).

### 3.1. Primary Outcomes

The change in weight-for-age z-score from HV1 to HV2 was significantly higher in RG compared to CG (effect size: 0.4 Z-scores; 95% CI: 0.09, 0.71), but the change in stress score over the 4 weeks was not significantly different (effect size: −0.2, 95% CI −2.8, 2.4; Table 3).

### 3.2. Secondary Outcomes

There were no significant differences between groups in stress score (effect size: −1.7; 95% CI: −4.3, 0.9; Table 3) or depression (effect size: −0.3; 95% CI: −2.4, 1.8; Table 4) at 6–8 weeks. Attachment and responsiveness to cues were also not different between groups. There were no significant differences in cortisol levels between groups (RG: 0.18 ± 0.07 vs. CG: 0.16 ± 0.08; *p* = 0.5). However, cortisol increased over time in the CG and decreased in the RG, and this change was significantly different (effect size: −0.08 ug/dL, 95% CI −0.15, −0.01). Verbal learning was also significantly higher (i.e., better) in the RG compared to the CG at 6–8 weeks (effect size: 1.1 words, 95% CI 0.04, 2.1; Table 4).

In the infants, there were no significant differences at 6–8 weeks in weight z-score (Table 3), length z-score (CG: 1.1 ± 1.3, n = 27 vs. RG: 1.2 ± 1.0, n = 28; *p* = 0.9), or change in length z-score (CG: 0.5 ± 0.8, n = 27 vs. RG: 0.3 ± 0.9, n = 28; *p* = 0.4) between groups. Median infant crying duration was significantly shorter in the RG compared to the CG [RG: 5.0 min, 0.0–120.0 vs. CG: 30.0 min, 0.0–142.0; *p* = 0.03] at 6–8 weeks, although this could be partially explained by the non-significantly shorter duration of crying in the RG at baseline [RG: 2.5 min, 0.0–190.0 vs. CG: 21.0 min, 0.0–172.0; *p* = 0.3]. No significant differences were found in any other behavior or appetite trait (all *p* > 0.05). These analyses were limited by the small number of participants completing the behavior diaries especially in the CG (55% of RG, 36% of CG).

Mothers in the RG breastfed significantly more times per day compared to those in the CG (effect size: 3.9; 95% CI: 0.9, 6.9; *p* = 0.01). There were no significant differences in fat, protein and carbohydrate concentrations in breast milk nor in estimated breast milk intake between groups (Table 5). These analyses were also limited by the small number of participants completing the procedures to assess breast milk intake, especially the test-weighing method, where 27% of RG and 25% of CG completed the breastfeeding diary and 9% of RG and 11% of CG completed the test-weighing.

### 3.3. Further Exploratory Analysis

Primary and secondary outcomes that were significantly different between the RG and CG remained significant after adjusting for sex and gestational age. No interactions between the intervention assignment and gestational age, sex, and recruitment during COVID-19 were found for the primary outcomes. The interactions found for the secondary outcomes are discussed in Supplementary File S1.

### 3.4. Dose-Response Associations

Only 23% of the participants complied with the suggested frequency of use and listened to the intervention 14 times or more, while 29% used it 3–13 times and the remainder less than 3 times. Some RG participants anecdotally reported using alternative relaxation therapies but did not record the frequency of use. Spearman’s correlations were used to assess the association between total times listened and the primary and secondary outcomes of the intervention. A positive and significant correlation was found between relaxation intervention use and weight z-score change (r = 0.31, *p* = 0.01), food responsiveness change (r = 0.32, *p* = 0.02) and breastfeeding frequency (r = 0.6. *p* = 0.008) at follow-up. Intervention use was also negatively associated with crying/colic duration (r = −0.5, *p* = 0.004) at follow-up. There were non-significant trends towards an association between intervention use and a decrease in cortisol (r = 0.2, *p* = 0.16), verbal learning (r = 0.24, *p* = 0.08), verbal forgetting (r = −0.20, *p* = 0.14), and protein concentration (r = −0.23, *p* = 0.09) at HV2.

## 4. Discussion

This study demonstrated that a simple maternal relaxation intervention was beneficial in promoting infant weight gain in a group of late preterm and early term infants. It also reduced maternal cortisol, decreased infant crying duration, improved verbal learning, and increased breastfeeding frequency.

The most robust finding is that the relaxation intervention was successful in improving infant growth as evidenced by the significantly higher and clinically meaningful weight-for-age z-score gain. It is generally recommended that nutritional interventions targeting preterm infants should aim to match the growth rate of a fetus of the same postmenstrual age [32]. Due to their larger size, and their reduced need for enteral/parenteral nutritional intervention compared to infants born at lower gestational ages, the exact nutrient requirements of late preterm infants, and thus the optimal growth patterns, are unknown. An acceptable target for LPI is estimated to be around 15 g/kg/day [32], but it remains unclear whether ETI have higher nutritional needs or should have higher growth velocities than later term infants. In this study, infants in the RG had significantly higher growth velocity despite the lack of direct nutritional intervention in these infants. While the weight gain (SDS = 0.77) achieved by infants in the RG might be considered rapid growth (often defined as SDS > 0.67) in the case of a healthy term infant [33], higher growth velocity is expected in these groups and the growth velocity achieved by these infants was closer to the recommendations mentioned above than that of infants in the CG. Additionally, although not measured in this study, the main component of the weight gain might be lean tissue rather than fat mass as suggested by a similar previous relaxation study involving full-term infants [16].

The intervention may also have affected stress favorably since cortisol, often considered a biomarker of stress, decreased significantly more in the RG. This is in line with previous studies reporting that relaxation therapies in breastfeeding mothers reduce perceived anxiety/stress and cortisol levels [34,35]. However, contrary to previous studies, our study did not demonstrate significant differences in stress and depression levels as reported by the participants. It could be that the reduction of cortisol was due to the higher frequency of breastfeeding observed in the intervention group irrespective of the effect of the intervention on stress. For instance, a previous study found that longer suckling durations were associated with lower cortisol levels [36]. It could also be due to the effects of the pandemic on the perception of stress levels and coping, where the usefulness or validity of the stress scale may have been different during the pandemic period. Lastly, it is possible that the reduction in cortisol level over time explains the better verbal learning scores observed in mothers in the intervention group. It was previously shown that in pregnant and postpartum women cortisol levels are associated in an inverted U-function with verbal recall scores [37,38,39].

We found that the duration of infant crying was significantly shorter in the intervention group compared to the control group. The shorter duration of crying could have potentially contributed to the increased infant weight gain observed in the intervention group. It is estimated that crying increases energy expenditure by ~5.4 times compared to quiet awake state [40] and it was also shown that general fussy behavior correlates with total daily energy expenditure [41]; all of which could incur a cost on growth. The pathway by which the intervention could have resulted in shorter crying duration could be related to nutrient provision. While more intense signaling might result in increased provisioning [42,43,44,45], increased feeding frequency seen in the intervention group could reflect “responsive feeding” and might decrease crying duration. The increased feeding frequency could also reflect increased breast milk volume or exclusivity, where fewer feeds might indicate supplementation with formula. However, the main limitation for infant behavior outcomes in this study was the small sample of participants completing the infant behavior diary and thus the results should be interpreted with caution.

From an evolutionary perspective, this study provides evidence for Trivers’ theory of parent-offspring conflict using an experimental approach. The theory predicts that, due to sharing only 50% of their genes, each infant is selected to demand more resources than its mother is selected to provide, resulting in tension over the magnitude of maternal metabolic resources allocated to the infant [17]. Psychological distress may raise such tension, potentially increasing maternal energy expenditure and influencing the metabolism of nutrients, which in turn may reduce the maternal energy budget for investment in the infant. In this study, reduction in stress could have led to the diversion of energy from stress to investment in the offspring through breastfeeding, by enabling greater transfer of breast milk and/or by impacting the infant via breast milk hormones. These hormones have the potential to promote infant growth directly, for example by promoting tissue growth, or indirectly by influencing infant behavior. Interestingly, we were also able to demonstrate that reducing mother-infant tension does not benefit the infant only, but also the mother by diverting energy away from stress and towards investment in her own energy capital as shown by better verbal memory.

### Limitations

There are several limitations that should be considered when interpreting the results. Low compliance with listening to the relaxation technique is a major limitation, where the participants used the intervention fewer times than advised. However, some participants in the RG reported using alternative therapies but did not record the frequency of use, and thus it was not possible to formally analyze the differences between those who frequently used relaxation techniques vs. those who did not or who occasionally used any relaxation technique. Nevertheless, beneficial effects were seen despite the low compliance. Another limitation is the relatively small sample size overall but also specifically for breast milk volume, where a small number of participants completed the test-weighing procedure. Due to this, we had to rely on the 24-h breastfeeding diary which was completed more frequently to estimate breast milk volume. The estimation was not based on a validated method, so it is possible that that the numbers are not accurate. Ideally, deuterium should have been used as it is the gold standard for estimating breast milk intake. However, it was expected that many mothers would not be exclusive breastfeeding at the breast, and thus it would have yielded inaccuracies in the estimation. Lastly, as per protocol, we did not adjust the *p*-value for multiplicity, therefore, the possibility of a type 1 error should be considered when interpreting the findings.

## 5. Conclusions

Our findings highlight the benefits of reducing maternal stress, since the relaxation intervention influenced infant growth and behavior as well as breastfeeding frequency. These results have scientific implications and contribute to the understanding of mother-infant signaling through breastfeeding. They also have clinical implications and suggest that simple relaxation tools should be considered for use in clinical settings; this is especially true for situations where the mother experiences high levels of stress or anxiety, and where there is concern about appropriate infant growth, such as in mothers of infants who have low birth weight or who are admitted to the NICU.

## Figures and Tables

**Table 1:** Baseline maternal and infant characteristics in whole sample and according to randomized group.

	Whole Sample (n = 69)	Control (n = 35)	Relaxation (n = 34)
Maternal age, years (mean ± SD)	33.1 ± 4.9	34.0 ± 4.8	32.3 ± 5.0
Infant gestation, weeks (mean ± SD)	36.5 ± 1.0	36.7 ± 1.1	36.3 ± 0.9
Birth weight, kg (mean ± SD)	2.6 ± 0.4	2.7 ± 0.4	2.6 ± 0.4
Male infant (n, %)	42 (60.9)	19 (54.3)	23 (67.6)
Primiparous (n, %)	47 (68.1)	23 (65.7)	24 (70.6)
Breastfeeding plan, months (mean ± SD)	9.7 ± 4.7	9.9 ± 5.8	9.4 ± 3.4
Late Preterm Infants ^1^ (n, %)	48 (69.6)	22 (62.9)	26 (76.5)
Maternal Ethnicity (n, %)			
White	38 (55.1)	19 (54.3)	19 (55.9)
Mixed/Multiple ethnic groups	2 (2.9)	1 (2.9)	1 (2.9)
Black/African/Caribbean/Black British	11 (15.9)	7 (20.0)	4 (11.8)
Asian/Asian British	12 (17.4)	5 (14.3)	7 (20.6)
Arab	3 (4.3)	1 (2.9)	2 (5.9)
Other ethnic group	3 (4.3)	2 (5.7)	1 (2.9)
Maternal education (n, %)			
≤5 GCSE A-C grade	3 (4.2)	1 (3.2)	2 (6.3)
A levels//equivalent	10 (14.5)	5 (16.1)	5 (15.6)
Bachelor’s degree	25 (36.2)	11 (35.5)	14 (43.8)
Master’s degree	15 (21.7)	11 (35.5)	4 (12.5)
PhD/professional qualification	10 (14.5)	3 (9.7)	7 (21.9)
Marital Status (n, %)			
Married/Civil Partnership/Cohabitation	55 (79.7)	29 (87.9)	26 (78.8)
Single parent- living on own	4 (5.8)	2 (6.1)	2 (6.1)
Single parent- living with family	6 (8.7)	1 (3.0)	5 (15.2)
Divorced	1 (1.4)	1 (3.0)	0 (0.0)
Household income (n, %)			
<£20–30 K	18 (28.6)	8 (26.7)	10 (30.3)
<£45 K–75 K	14 (22.2)	8 (26.7)	6 (18.2)
<£100 K	12 (19.0)	6 (17.1)	6 (18.2)
>£100 K	19 (30.2)	8 (26.7)	11 (33.3)

^1^ Despite stratification according to gestational age, there was a slight imbalance in the percent late preterm vs. early term in the randomized groups. This is likely to be due the three participants who were assigned randomization numbers but then did not provide any data.

**Table 2:** Differences in baseline hospital practices between randomized groups.

	Control Group (n = 35)	Intervention Group (n = 34)
Did not attend breastfeeding classes (n, %)	22 (62.9)	23 (67.6)
Type of Delivery (n, %)		
Vaginal- not induced	10 (28.6)	12 (35.3)
Vaginal- induced	8 (22.9)	5 (14.7)
C-section- planned or elective	3 (8.6)	2 (5.9)
C-section- emergency or unplanned	14 (40.0)	15 (44.1)
Skin-to-Skin: How Soon (n, %)		
Did not have skin-to-skin	5 (14.3)	8 (23.5)
Directly	16 (45.7)	16 (47.1)
Within 30 min after birth	4 (11.4)	4 (11.8)
More than 30 min after birth	6 (17.1)	4 (11.8)
More than 1 h after birth	4 (11.4)	2 (5.9)
Skin-to-Skin: How Long (n, %)		
Did not have skin-to-skin	5 (14.3)	8 (23.5)
Less than 30 min	14 (40.0)	16 (47.1)
Between 30 to 60 min	8 (22.9)	4 (11.8)
More than 1 h	8 (22.9)	6 (17.6)
Breastfeeding Initiation: How Soon (n, %)		
Less than 30 min after birth	6 (17.1)	9 (26.5)
More than 30 min after birth	29 (82.9)	25 (73.5)
Mother and Infant Rooming-In (n, %)		
Did not room-in at all	2 (5.7)	3 (8.8)
Sometimes	6 (17.1)	7 (20.6)
At all times	27 (77.1)	24 (70.6)
Hospital Stay		
Admitted to NICU (n, %)	7 (20.0)	11 (32.4)
Length of stay at NICU (median [range])	5.0 [1,2,3,4,5,6,7,8,9,10,11,12,13,14]	5.0 [1,2,3,4,5,6,7,8,9,10,11,12,13,14,15,16,17,18,19,20,21,22,23,24,25,26,27,28,29,30,31,32,33,34,35,36,37]
Length of hospital stay (median [range])	4.0 [1,2,3,4,5,6,7,8,9,10,11,12,13]	4.0 [1,2,3,4,5,6,7,8,9,10,11,12,13,14,15,16,17,18,19,20,21,22,23,24,25,26,27,28,29,30,31,32,33,34,35,36,37]
Supplementation		
Did not supplement infant formula (n, %)	5 (14.3)	12 (35.3)
<30 mL per feed, on average (n, %)	21 (60.0)	13 (38.2)
30–60 mL per feed, on average (n, %)	9 (25.7)	9 (26.5)
Times/day expressed breast milk (mean ± SD)	2.5 ± 3.8	2.8 ± 3.4
Support		
Offered support for feeding problems	28 (80.0)	26 (76.5)
Received enough help with feeding	20 (58.8)	23 (67.6)
Given details of community support groups	30 (85.7)	32 (94.1)

**Table 3:** Differences in the primary outcomes of the relaxation intervention.

		Control Group		Relaxation Group	*p*-Value ^1^	MD	95% CI
Primary Maternal Outcomes	n	Mean ± SD	n	Mean ± SD			
Perceived Stress HV1 ^2^	31	15.2 ± 5.2	33	14.8 ± 5.8	0.8	−0.4	−3.2, 2.4
Perceived Stress HV2	34	13.7 ± 5.0	33	12.0 ± 5.5	0.2	−1.7	−4.3, 0.9
Change Stress	30	−2.7 ± 5.0	32	−2.9 ± 5.3	0.9	−0.2	−2.8, 2.4
Primary Infant Outcome							
Weight Z-Score HV1	35	−0.32 ± 0.95	35	−0.37 ± 0.89	0.9	−0.09	−0.50, 0.40
Weight Z-Score HV2	35	0.07 ± 0.97	34	0.43 ± 0.79	0.1	0.37	−0.06, 0.80
Change Weight Z-Score	33	0.37 ± 0.61	34	0.77 ± 0.66	0.01	0.40	0.09, 0.71

^1^ Independent sample *t*-test was used to test the differences between the randomized groups. ^2^ HV: home visit; HV1 took place at 2–3 weeks and HV2 took place at 6–8 weeks.

**Table 4:** Comparison of depression, responsiveness to cues, attachment and verbal memory scores between randomized groups.

		Control Group		Relaxation Group	*p*-Value ^1^	MD	95% CI
Psychological State	n	Mean ± SD	n	Mean ± SD			
Depression score HV1 ^2^	27	7.6 ± 4.7	31	7.8 ± 4.4	0.8	0.2	−2.2, 2.6
Depression score HV2	30	6.5 ± 3.8	31	6.2 ± 4.2	0.8	−0.3	−2.4, 1.8
Change in Depression score	24	−2.0 ± 3.2	30	−1.5 ± 3.4	0.6	0.5	−1.3, 2.3
Salivary Cortisol (ug/dL)							
Cortisol HV1	25	0.15 ± 0.07	26	0.22 ± 0.13	0.03	0.07	0.01, 0.13
Cortisol HV2	24	0.16 ± 0.08	25	0.18 ± 0.07	0.5	0.02	−0.02, 0.06
Change in Cortisol	20	0.05 ± 0.13	21	−0.03 ± 0.10	0.03	−0.08	−0.15, −0.01
Responsiveness and Attachment							
Responsiveness HV1	25	4.3 ± 0.5	28	4.2 ± 0.5	0.7	−0.1	−0.4, 0.2
Responsiveness HV2	22	4.2 ± 0.3	27	4.2 ± 0.4	0.7	0.0	−0.2, 0.2
Delayed Responsiveness HV1	25	2.3 ± 0.9	28	2.5 ± 0.7	0.5	0.2	−0.2, 0.6
Delayed Responsiveness HV2	22	2.7 ± 0.9	27	2.5 ± 0.7	0.5	−0.2	−0.7, 0.3
Non-Responsiveness HV1	25	1.1 ± 0.3	28	1.1 ± 0.3	0.7	−0.04	−0.2, 0.1
Non-Responsiveness HV2	22	1.1 ± 0.2	27	1.0 ± 0.1	0.2	−0.05	−0.1, 0.03
Maternal Attachment HV1	23	70.9 ± 8.1	30	71.6 ± 6.4	0.7	0.7	−3.3, 4.7
Maternal Attachment HV2	21	71.9 ± 4.2	27	73.2 ± 4.4	0.3	1.2	−1.3, 3.8
Verbal Memory (words)							
Immediate Recall HV1	31	54.3 ± 7.0	30	56.1 ± 5.5	0.3	1.8	−1.4, 5.0
Immediate Recall HV2	25	59.2 ± 6.3	28	61.4 ± 6.2	0.2	2.2	−1.3, 5.6
Verbal Learning HV1	31	5.8 ± 1.9	30	6.1 ± 1.8	0.6	0.3	−0.6, 1.3
Verbal Learning HV2	25	3.5 ± 1.9	28	4.6 ± 1.8	0.04	1.1	0.04, 2.1
Verbal Forgetting HV1	31	−1.7 ± 1.7	30	−1.3 ± 1.8	0.4	0.4	−0.5, 1.3
Verbal Forgetting HV2	25	−0.4 ± 1.6	28	−1.0 ± 1.0	0.1	−0.6	−1.3, 0.2

^1^ Independent sample *t*-test was used to test the differences between the randomized groups. ^2^ HV: home visit; HV1 took place at 2–3 weeks and HV2 took place at 6–8 weeks.

**Table 5:** Comparison of breastfeeding- and breastmilk-related outcomes between groups.

		Control Group		Relaxation Group	*p*-Value ^1^	MD	95% CI
Breast Milk Composition	n	Mean ± SD	n	Mean ± SD			
Fat HV1 ^2^ (g/100 mL)	30	4.0 ± 1.0	29	3.6 ± 1.0	0.1	−0.4	−0.9, 0.1
Fat HV2 (g/100 mL)	28	4.1 ± 1.1	28	4.0 ± 1.2	0.7	−0.1	−0.8, 0.5
Change Fat	25	0.05 ± 1.3	22	0.4 ± 1.6	0.5	0.3	−0.6, 1.2
Protein HV1 (g/100 mL)	30	1.2 ± 0.3	29	1.1 ± 0.2	0.03	−0.1	−0.2, −0.01
Protein HV2 (g/100 mL)	28	1.0 ± 0.2	28	0.9 ± 0.2	0.07	−0.1	−0.2, 0.01
Change Protein	25	−0.3 ± 0.2	22	−0.3 ± 0.1	0.8	0.01	−0.07, 0.1
Carbohydrates HV1 (g/100 mL)	30	7.2 ± 0.3	29	7.2 ± 0.4	0.4	0.08	−0.1, 0.3
Carbohydrates HV2 (g/100 mL)	28	7.2 ± 0.4	28	7.3 ± 0.3	0.4	0.08	−0.1, 0.3
Change Carbohydrates	25	0.04 ± 0.3	22	0.2 ± 0.4	0.07	0.2	−0.01, 0.4
Energy HV1 (kcal/100 mL)	30	72.8 ± 9.7	29	68.7 ± 8.7	0.09	−4.1	−8.9, 0.7
Energy HV2 (kcal/100 mL)	28	72.7 ± 10.1	28	71.2 ± 11.0	0.6	−1.5	−7.2, 4.2
Change Energy	25	−0.6 ± 12.6	22	2.9 ± 14.9	0.4	3.5	−4.5, 11.6
Direct Breast Milk Intake							
Volume-Test Weighing HV1	7	419 ± 270	10	347 ± 168	0.6	−72	−297, 154
Volume- Test Weighing HV2	4	439 ± 163	3	623 ± 194	0.2	184	−162, 530
Change Volume-Test Weighing	4	88 ± 451	1	320	0.7		
Volume- 24 h Diary HV1	11	501 ± 269	14	511 ± 251	0.9	10	−205, 226
Volume- 24 h Diary HV2	9	388 ± 250	9	519 ± 201	0.2	131	−96, 358
Change Volume- BF Diary	5	−125 ± 136	6	−0.5 ± 82	0.1	125	−25, 274
Feeding Patterns							
Breastfeeding Frequency HV1	13	7.5 ± 5.0	14	8.7 ± 2.5	0.4	1.2	−1.9, 4.3
Breastfeeding Frequency HV2	9	5.9 ± 3.0	9	9.8 ± 3.0	0.01	3.9	0.9, 6.9

^1^ Independent sample *t*-test was used to test the differences between the randomized groups. ^2^ HV: home visit; HV1 took place at 2–3 weeks and HV2 took place at 6–8 weeks.

## Data Availability

Deidentified individual participant data will be made available upon publication to researchers who provide a methodologically sound proposal for use in achieving goals of the approved proposal. Proposals should be submitted to sarah.dib@ucl.ac.uk.

## Supplementary Materials

The following are available online at https://www.mdpi.com/article/10.3390/nu14235041/s1, Table S1: CONSORT 2010 checklist of information to include when reporting a randomised trial; File S1: File S1. Discussion for the Interactions Found For the Secondary Outcomes.
